# Auxotrophy from bioenergetic demand: the case of proline addiction in *Clostridioides difficile*

**DOI:** 10.1128/msphere.00246-26

**Published:** 2026-06-26

**Authors:** Michael A. Johnstone, William T. Self

**Affiliations:** 1 Burnett School of Biomedical Sciences, College of Medicine, University of Central Florida 6243, Orlando, Florida, USA; The University of Iowa, Iowa City, Iowa, USA

**Keywords:** proline, proline reductase, auxotrophy, addiction, selenoprotein, Stickland reaction

## Abstract

Many microbial growth requirements are interpreted as auxotrophy arising from loss of biosynthetic capacity. *Clostridioides difficile* is widely considered to be proline auxotrophic because it fails to grow in defined media lacking proline, yet it harbors proline biosynthesis genes. Because *C. difficile* preferentially uses proline as an electron acceptor via D-proline reductase (Prd), we argue that this phenotype arises from a bioenergetic constraint rather than an inability to make proline. Consistent with this view, mutants lacking Prd gain the ability to grow without proline. Moreover, conditions that adapt the organism to Prd-independent redox metabolism, such as Wood-Ljungdahl-linked acetobutyrogenesis, also enable proline-independent growth. We propose a model for “proline addiction” in which sustained Prd flux depletes the intracellular proline pool faster than biosynthesis can replenish it, making growth dependent on environmental proline. This bioenergetic addiction model may help explain cryptic auxotrophies that genome-based cultivation approaches would otherwise miss.

## OPINION/HYPOTHESIS

Auxotrophy refers to the growth requirement for a specific nutrient, a concept historically rooted in the inability of cells to synthesize that nutrient. Since the mid-20th century, auxotrophic mutants have played a foundational role in defining the relationship between genes and metabolism. George Beadle and Edward Tatum—pioneers of the “one gene–one enzyme” hypothesis—used X-rays in 1941 to generate nutritional mutants of *Neurospora crassa* and *Neurospora sitophila* (1), providing evidence that specific genetic defects could interfere with metabolic pathways. The terminology itself emerged less than a decade later when Bernard Davis and Elizabeth Mingioli suggested the terms “auxotroph” and “auxotrophic,” derived from the Latin *auxilium* (“aid”) and Greek *trophē* (“food”), in their characterization of nutritional mutants of *Escherichia coli* requiring methionine or vitamin B_12_ (2). Joshua and Esther Lederberg later introduced replica plating as a rapid method to screen hundreds of colonies across multiple media (3), including minimal media used to identify auxotrophs. Bruce Ames further exploited auxotrophy as a powerful experimental readout in the Ames test, using *Salmonella typhimurium* histidine auxotrophs to detect mutagens by selecting for reversion mutations in histidine biosynthesis genes (4, 5). Together, these examples established auxotrophy as both a practical genetic tool and a phenotype strongly associated with defects in nutrient biosynthesis.

Beyond its utility as a genetic tool, auxotrophy is a physiological reality that manifests differently across microbial species. *Haemophilus influenzae* is a classic clinical example because it is unable to synthesize heme and NAD^+^ and therefore requires these factors exogenously (6). *Mycoplasma* species represent a more extreme case, often displaying multiple auxotrophies arising from extensive genome reduction (7). However, some auxotrophic phenotypes are conditional, arising despite the presence of intact biosynthetic genes. *Listeria monocytogenes*, for example, exhibits a controlled branched-chain amino acid (BCAA) auxotrophy in which CodY and the Rli60 riboregulator restrict BCAA biosynthesis, thereby preserving isoleucine as a signal for virulence gene activation (8). Similarly, despite encoding redundant proline biosynthesis pathways, *Staphylococcus aureus* can behave as a proline auxotroph with CcpA/HPr-mediated carbon catabolite repression contributing to the phenotype (9). Nutrient-dependent growth can also be shaped by ecological context. In 2002, Kaeberlein and colleagues successfully cultured “unculturable” environmental microbes from marine sediment using diffusion chambers with nutrient-permeable membranes (10). Many of these isolates failed to grow on petri dishes containing standard laboratory media unless co-cultured with neighboring microbes from the original sample, demonstrating that interactions within the native microbial community can impose growth requirements (10). Therefore, while auxotrophy is commonly associated with defects in nutrient biosynthesis, it is a growth phenotype that can arise through multiple mechanisms, including mechanisms that remain poorly characterized. Here, we call attention to one such mechanism by re-examining proline auxotrophy in the nosocomial pathogen *Clostridioides difficile* and describing it as a “bioenergetic addiction,” a type of conditional auxotrophy in which growth dependence is best explained by metabolic demand rather than a broken proline biosynthetic pathway.

*C. difficile* is a clinically significant pathogen that has affected the infectious disease landscape since its spread in the 1970s. *C. difficile* causes antibiotic-associated diarrhea and pseudomembranous colitis primarily through the actions of its exotoxins TcdA and TcdB (11). Management of a *C. difficile* infection (CDI) remains challenging, particularly in recurrent cases following antibiotic therapy. Fecal microbiota transplantation has proven highly effective for treating recurrent CDI, presumably by restoring microbial communities that restrict *C. difficile* colonization (11). Recent evidence has shown that colonization appears to hinge on acquisition of key gut nutrients, particularly the amino acid proline (12, 13), which was originally shown to be required for growth in a *C. difficile* minimal medium designed by Karasawa and colleagues in 1995 (14). Indeed, it is well known within the field that *C. difficile* cannot grow unless proline is supplied exogenously, which would initially suggest a biosynthetic deficiency. However, while *C. difficile* lacks the *proB* and *proA* genes associated with the canonical glutamate-to-proline pathway (15), it still contains two *proC*-like genes annotated as pyrroline-5-carboxylate (P5C) reductases (*proC1* [CD630_14950] and *proC2* [CD630_32810]), which can reduce P5C to L-proline. Since P5C cannot be generated through the canonical ProB/ProA route, hydroxyproline can instead provide P5C via the hydroxyproline dehydratase HypD (16). Additionally, *C. difficile* encodes a putative ornithine cyclodeaminase-family protein (CD630_05440) predicted to convert L-ornithine to L-proline and an entire set of ornithine biosynthesis genes (15, 17). Because the organism evidently has the genetic capacity to synthesize its own proline, its growth phenotype cannot be readily explained by classical biosynthetic auxotrophy. In *C. difficile*, however, proline participates in both biosynthesis and energy metabolism: L-proline contributes to protein synthesis, whereas D-proline serves as a preferred electron acceptor in Stickland metabolism, a bioenergetic process in which amino acids are coupled in paired redox reactions (18). These pools are linked by the proline racemase PrdF, which converts L-proline to D-proline; for simplicity, both forms are often referred to here as “proline” unless stereochemistry is relevant (18). The selenium-dependent D-proline reductase (Prd) then catalyzes the reduction of D-proline to 5-aminovalerate, a reaction that is coupled to NAD^+^ regeneration (18). Because collagen-derived Stickland acceptors such as proline and hydroxyproline are abundant in the gut during infection (19), Prd-dependent proline reduction likely provides a bioenergetic advantage during colonization. In support of this view, *prdB* mutants exhibit impaired colonization and reduced or delayed toxin production in humanized microbiota-associated and gnotobiotic mouse models (12, 20). However, this still does not resolve the central paradox: why does *C. difficile* require exogenous proline for growth despite apparent biosynthetic capacity?

**Fig 1:**
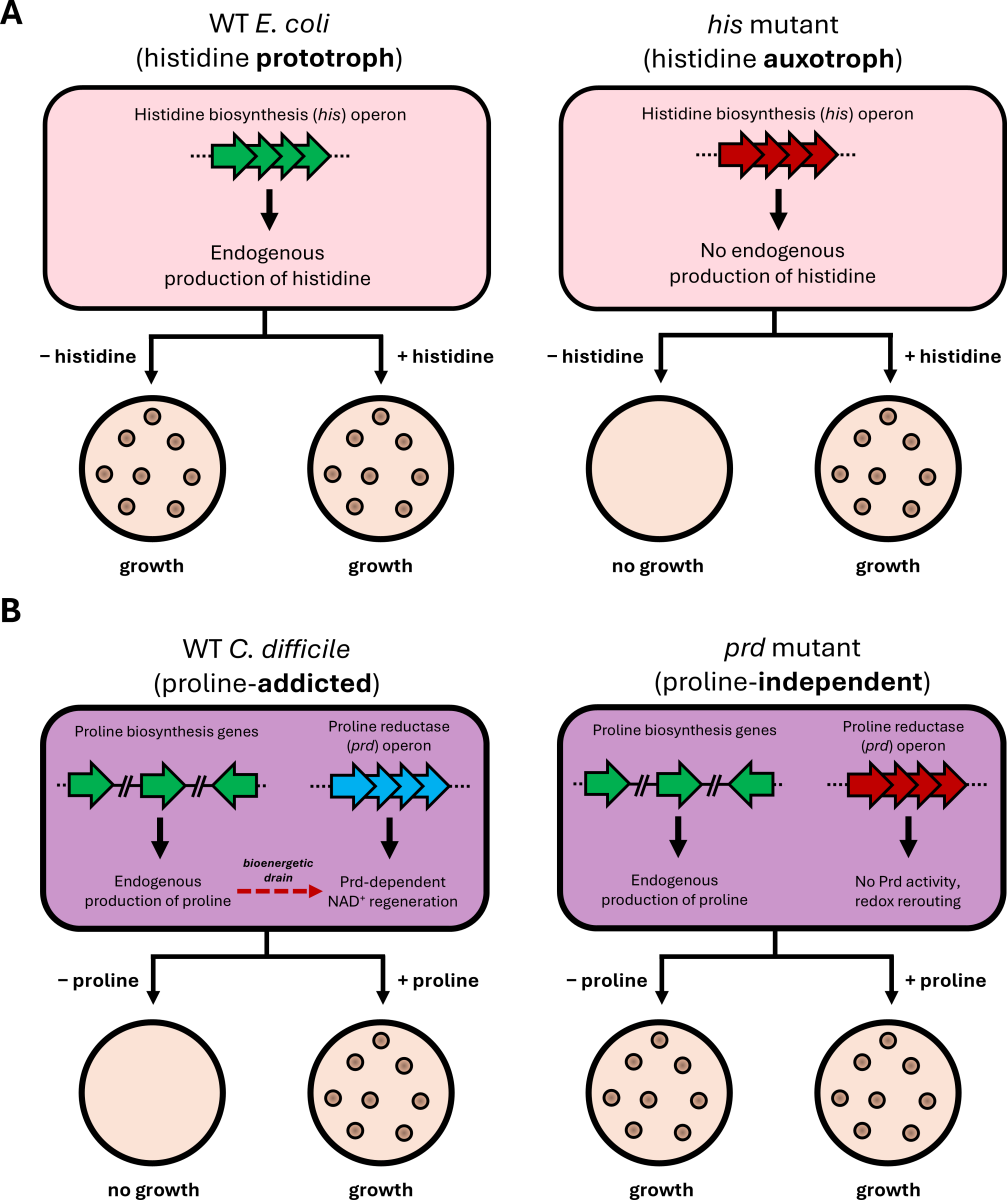
A model comparing *E. coli* histidine prototrophy and auxotrophy with *C. difficile* proline addiction. (**A**) (Left) Wild-type *E. coli* exhibits histidine prototrophy due to the presence of histidine biosynthesis genes found in the *his* operon (green arrows). The *his* operon encodes the machinery required to produce intracellular histidine, allowing for growth independent of exogenous histidine (represented by colonies on an agar plate). (Right) By contrast, *E. coli* mutants with a defective *his* operon (red arrows) fail to synthesize their own histidine and are therefore rendered auxotrophic, resulting in an inability to grow without supplemented histidine (no colonies on plate). (**B**) (Left) Wild-type *C. difficile* exhibits proline auxotrophy despite containing proline biosynthesis genes (green arrows, found in different locations in the genome). Wild-type cells therefore cannot grow unless exogenous proline is present. *C. difficile* also contains the *prd* operon (blue arrows), which encodes the D-proline reductase (Prd), a selenoenzyme that couples proline reduction to NAD^+^ regeneration. The presence of Prd likely imposes a bioenergetic drain on the pool of intracellular proline, effectively preventing the organism from supporting its own growth in proline-limited conditions. (Right) By contrast, *C. difficile* cells with mutations in *prd* genes (red arrows) gain the ability to grow independently of exogenous proline. In these mutants, the loss of Prd-dependent NAD^+^ regeneration is presumably offset by alternative Prd-independent redox pathways, thereby relieving the bioenergetic drain on the proline pool and allowing endogenous proline to contribute more to biomass and support growth.

In 2022, we investigated the effects of collagen-derived Stickland acceptors—glycine, proline, and hydroxyproline—on the growth and physiology of several *C. difficile* mutant strains unable to perform selenium-dependent Stickland reactions (21). During growth experiments in minimal media with or without these amino acids, we found that mutants deficient in selenophosphate synthetase (*selD*), the central enzyme required for selenoprotein production, could surprisingly grow in the absence of supplemented proline (21). We reasoned that the *selD* phenotype was likely attributed to the loss of Prd, which itself is a selenoprotein. Indeed, we discovered that strains deficient in Prd (via mutations in *prdB*, the selenium-containing subunit, or *prdR*, the transcriptional regulator) also gained the ability to grow in proline-deficient media (21). This finding was later observed in 2024 by Behlendorf and colleagues, who reported that the growth of a mutant lacking the Prd maturation enzyme PrdH was insensitive to proline concentrations (22). Thus, when the organism lost its apparent ability to utilize its preferred electron acceptor, it simultaneously lost its requirement for exogenous proline. We propose that Prd-dependent proline reduction drains the intracellular proline pool faster than it can be replenished by biosynthesis, making exogenous proline necessary for growth. When Prd is removed, this drain disappears, and the organism can now grow without external proline. These findings indicate that the proline dependency of *C. difficile* is a type of conditional auxotrophy: rather than reflecting an inability to synthesize proline, it appears to reflect a strict bioenergetic preference that is self-limiting in proline-poor environments. We refer to this auxotrophic phenomenon as “proline addiction” because the growth requirement appears to arise from preferred use of proline for redox balance rather than from loss of proline biosynthetic capacity (Fig. 1). At first glance, the self-limiting nature of proline addiction does not appear to be advantageous for growth, as this cost becomes apparent under proline-deficient minimal medium conditions. However, in the infected gut, which is rich in collagen-derived proline and hydroxyproline, environmental supply may satisfy this bioenergetic demand, allowing *C. difficile* to prioritize proline reduction as an efficient redox-balancing pathway without imposing the same growth limitation.

We previously hypothesized that the disappearance of the proline-addicted phenotype was due to a compensatory shift in redox metabolism following the loss of Prd-dependent NAD^+^ regeneration (21). One plausible route is acetobutyrogenesis, the coupling of the Wood-Ljungdahl pathway (WLP) to butyrate formation described by Gencic and Grahame in 2020 (15). In that study, the authors noted that the WLP was generally inactive in peptide-rich media, which are replete with Stickland acceptors such as proline, leucine, and glycine, and therefore had to be studied under minimal conditions where these acceptors were absent. Direct culturing in minimal media lacking proline or leucine did not support *C. difficile* growth, so the authors progressively adapted the organism to glucose minimal medium with decreasing concentrations of these Stickland acceptors in order to promote WLP utilization (15). This stepwise transition enabled growth in glucose minimal medium without Stickland acceptors, including proline, supporting the idea that proline dependence in *C. difficile* is conditional and related to bioenergetics rather than biosynthesis. Although the genetic basis of this adaptation was not defined, the authors interpreted the adapted phenotype as a physiological transition toward WLP-linked acetobutyrogenesis, further theorizing that low flux through the WLP paired with butyrate metabolism would satisfy redox requirements and support highly efficient ATP production from glucose oxidation. Together, these observations strengthen the argument that proline-free growth can occur when redox balance is achieved through a Prd-independent mechanism. Whether Prd-deficient mutants rely on WLP-driven acetobutyrogenesis or on a different compensatory redox pathway remains unknown, especially because our growth conditions did not require complete withdrawal of all Stickland acceptors (21). Consistent with this uncertainty, recent *in vivo* transcriptomic analysis of a Δ*prdB* mutant revealed broad changes in Stickland metabolism, carbohydrate utilization, mixed-acid fermentation, and energy-conserving systems such as the Rnf complex, V-type ATP synthases, and hydrogenases, suggesting that Prd-deficient cells may use multiple compensatory routes rather than a single replacement pathway (20). Nevertheless, the shared ability of adapted wild-type cells and Prd-deficient mutants to grow without exogenous proline supports the broader idea that proline dependence is conditional and tied to bioenergetics.

The field of microbiology has greatly evolved since the early days of replica plating with auxotrophic mutants. Today, cultivation of unknown microbes often relies heavily on genome-based predictions of essential nutrients inferred from annotated biosynthetic pathways. Although this approach is useful in developing and optimizing growth media, it often misses ecological and metabolic constraints that shape growth (23, 24). If *C. difficile* had been approached today using genome-guided medium design, its proline biosynthesis genes might have suggested that proline is not required. Here, we present proline addiction as a case study illustrating why biosynthetic gene content alone does not always account for a growth phenotype. Our model proposes that the apparent proline requirement in *C. difficile* arises because Prd flux imposes a bioenergetic drain on the intracellular proline pool through sustained proline reduction (Fig. 1). Future studies should be designed to test the assumptions in this model: (i) wild-type cells should have lower intracellular proline and/or higher proline turnover relative to Prd-deficient cells (e.g., *selD* and *prd*) under proline-limiting conditions; and (ii) experimentally tuning Prd activity (genetic knockdown/overexpression or chemical inhibition) should tune the severity of the addiction phenotype. Practically, this model suggests that genome-guided cultivation should consider not only whether a nutrient can be biosynthesized, but also whether that nutrient can be consumed by high-flux pathways involved in redox balance or energy conservation. Genome-based culturing methods should therefore account for bioenergetic addiction as a possible explanation when nutrient requirements persist despite predicted biosynthetic capacity. If the proline auxotrophy of one of our most clinically significant pathogens is best explained by bioenergetic addiction rather than a classical biosynthetic defect, how many other auxotrophies have we misunderstood?
